# Toxic and hormetic-like effects of three components of citrus essential oils on adult Mediterranean fruit flies (*Ceratitis capitata*)

**DOI:** 10.1371/journal.pone.0177837

**Published:** 2017-05-16

**Authors:** Stella A. Papanastasiou, Eleftheria-Maria D. Bali, Charalampos S. Ioannou, Dimitrios P. Papachristos, Kostas D. Zarpas, Nikos T. Papadopoulos

**Affiliations:** 1 Department of Agriculture, Crop Production and Rural Environment, University of Thessaly, Nea Ionia, Magnisia, Greece; 2 Department of Entomology and Agricultural Zoology, Benaki Phytopathological Institute, Kifissia, Athens, Greece; University of Crete, GREECE

## Abstract

Plant essential oils (EOs) and a wide range of their individual components are involved in a variety of biological interactions with insect pests including stimulatory, deterrent, toxic and even hormetic effects. Both the beneficial and toxic properties of citrus EOs on the Mediterranean fruit fly (medfly) have been experimentally evidenced over the last years. However, no information is available regarding the toxic or beneficial effects of the major components of citrus EOs via contact with the adults of the Mediterranean fruit fly. In the present study, we explored the toxicity of limonene, linalool and α-pinene (3 of the main compounds of citrus EOs) against adult medflies and identified the effects of sub-lethal doses of limonene on fitness traits in a relaxed [full diet (yeast and sugar)] and in a stressful (sugar only) feeding environment. Our results demonstrate that all three compounds inferred high toxicity to adult medflies regardless of the diet, with males being more sensitive than females. Sub-lethal doses of limonene (LD_20_) enhanced the lifespan of adult medflies when they were deprived of protein. Fecundity was positively affected when females were exposed to limonene sub-lethal doses. Therefore, limonene, a major constituent of citrus EOs, induces high mortality at increased doses and positive effects on life history traits of medfly adults through contact at low sub-lethal doses. A hormetic-like effect of limonene to adult medflies and its possible underlying mechanisms are discussed.

## Introduction

Host (and non-host) plant essential oils and/or numerous of their individual components are involved in a wide range of biological phenomena of insect herbivores [1]. These may include stimulatory, deterrent, toxic and even hormetic effects [2–5]. Essential oils (EOs) and other secondary metabolites constitute components of the plant defensive mechanisms and strong drivers of evolutionary events inducing selection pressures to herbivores [6].

The Mediterranean fruit fly (medfly), *Ceratitis capitata* (Wiedemann) (Diptera: Tephritidae) is considered one of the most important insect pests for the production and trading of fresh fruits and vegetables [7]. It is a multivoltine, cosmopolitan and an extremely polyphagous species infesting more than 350 plant species [8, 9]. Within the long list of medfly hosts, citrus fruits (Rutaceae) stand out. The rind of citrus fruit contains a dense layer of essential oil glands that are involved in fruit defense against many frugivorous insects, such as medfly, and fungi. The interaction between medfly and citrus fruits from different species and cultivars has been explored since the beginning of the 20^th^ century. Back and Pemberton [10] have first demonstrated the defensive properties of citrus fruit against medfly infestation, which have been further explored by Rivnay [11]. Later, in 1980s, Roessler [12] has proposed the use of gibberellic acid to modify the chemical properties of citrus ripening fruit towards conferring resistance against medfly infestation in field conditions. Over that last few decades, following advances in analytical chemical methodologies, the beneficial and toxic properties of citrus EOs against medfly have been further explored [13]. Toxic properties of the citrus rind on medfly larvae have been demonstrated by Salvatore et al. [3] and by Papachristos et al. [14]. The toxicity of citrus oils against medfly larvae is related to quantitative and qualitative aspects of their principal components (monoterpenes and sesquiterpenes). Citrus EOs contain mainly limonene (> 90% in some species), which causes moderate toxicity against medfly larvae compared to other components of citrus oils [14]. Oxygenated compounds of the citrus fruit, such as linalool, were found to be more toxic compared to non-oxygenated terpenes (limonene) followed by the least toxic α- and β-pinenes.

Accumulation of scientific evidences regarding the relationships between plant secondary metabolites and insect herbivores, especially those involving toxic properties, advances in analytical and synthetic chemistry, high throughput screening systems as well as the need for safer insecticides compounds have brought EOs and other natural products on the spot for developing novel pest control strategies. Along these lines, several EOs have been tested in laboratory conditions amongst others against adult Mediterranean fruit flies. For example, Miguel et al. [15] and Chang et al. [16] respectively reported fumigant toxic properties of *Mentha pulegium*, and basil EOs and their major components linalool, trans-anethole, and estragole on adult medflies. The ingestion of extracts from *Thymus herba-barona* and *Cinnamomum zeyanicum* (both rich in phenolics and aromatic aldehydes) have been shown to induce high mortality rates on adult *C*. *capitata* while that of *Salvia officinalis* and *Rosmarinus officinalis* (rich in monoterpenic ketones and monoterpene hydrocarbons) much lower [17]. EOs from *Bacharis darwinii* (containing high levels of limonene, and much lower in thymol and 4-terpineol) [18] and *Tagetes* spp (rich in oxygenated monoterpenoids) [19] have shown substantial toxicity on adult medflies following topical application. A few additional recent studies have revealed fumigant, injection and contact toxic properties of *Melaleuca alternifolia* (rich in monoterpene hydrocarbons and oxygenated monoterpenes) [20], *and Rosmarinus officinalis*, *Lavandula angustifolia*, *Hyptis suaveolens and Thuja occidentalis* [21]. Moving a step forward from the standard laboratory toxicity tests Arancibia et al. [22] have developed biodegradable films to deliver clove and citronella oils against medflies. Citronella oil and substantial vapor by films containing clove oil exhibited high toxic properties and induced mortality to adult medflies.

Stepping on previous knowledge regarding beneficial and toxic properties of citrus EOs on adult Mediterranean fruit flies, and considering the above efforts of many research groups to develop pesticides from natural products the current study provides additional experimental evidences for the contact toxic properties of three major components of citrus EOs—limonene, linalool and α-pinene—on adult medflies. In addition, we have explored the effects of sub-lethal doses of limonene (the most abundant constituent of citrus EOs) on adult life history traits, such as longevity and female fecundity.

## Materials and methods

### Experimental conditions and flies

The experiments were conducted at the Laboratory of Entomology and Agricultural Zoology, University of Thessaly, Greece, in standard conditions (25 ± 1°C, 65 ± 5% R.H., and a photoperiod of L14:D10 with photophase starting at 07.00 h.). The medflies used in our experiments were from the laboratory strain “Benakeio” which has been maintained under laboratory conditions for more than 30 years and were reared following the protocol described by Diamantidis and co- workers [23].

Upon emergence adults were placed until the testing dates in 20 by 20 by 20 cm Plexiglas cages (maximum 20 individuals per cage). Following toxicity bioassays to determine the LD_50_ of the 3 compounds of citrus EOs, adults were placed in 400 ml plastic transparent caps in groups of 5. After the application of the sub-lethal doses (LD_20_) of limonene aiming to explore effects on life history traits, adults were individually placed in cages (plastic transparent caps—see above) with those designated for females possessing an artificial oviposition substrate [5 cm diameter hollow, plastic red coloured hemisphere (dome) bearing 40–50 evenly distributed holes (1 mm diameter)]. Additional details regarding individual cages and oviposition devices are given in Sarakatsanou et al. [24]. All flies had free access to water and adult food [(yeast hydrolysate, sugar and water at 4:1:5 ratio (YS), or sugar and water at 1:3 ratio (S)].

### Toxicity bioassay

We tested the toxic effects of 3 components of citrus essential oil: linalool (97% purity), (-)-α-pinene (98% purity) and (R)-(+)-limonene (97% purity) (Sigma Aldrich^®^). The susceptibility of medfly adults to the major components of citrus EOs was evaluated using a micro-drop bioassay. The procedure we followed was similar for all experiments. In detail, each compound was either used in its pure form (100%) or dissolved and diluted to eight different concentrations (0.5, 1.0, 1.5, 2.0, 2.5, 3.0, and 25%) with acetone (> 99.5% purity, Sigma Aldrich^®^). Using an aspirator, 5–7 days old flies were transferred from the Plexiglas cages to a CO_2_ anaesthetization device. Anesthetization was accomplished by exposure to a light stream of CO_2_ that lasted less than 30 seconds. Once immobile, a droplet (2 μl) of the solution was applied topically onto the abdomen of each fly using a hand, borosilicate glass micro-syringe (Witeg^®^ Germany). Control flies were treated with the same quantity of acetone alone. After the bioassay, flies were transferred in groups of 5 to the individual plastic cages using soft forceps. The mortality was recorded 24 hours after the test. Flies were considered dead if appendages remained still when prodded with a fine entomological pin.

### Effect of limonene sub-lethal dose (LD_20_) and diet on adult demographic traits

The sub-lethal dose LD_20_ of limonene for each treatment (3.47 nl per male fed with S, 2.8 nl per male fed with YS, 12.26 nl per female fed with S and 11.15 nl per female fed with YS) was applied on 400 adults (100 males and 100 females fed with S, 100 males and 100 females fed with YS) at the age of 5 days, using a micro-drop bioassay as described above. Limonene was selected among the three compounds tested because (as mentioned above) it is the most abundant compound of citrus EOs [2, 14]. Subsequently, flies were sorted by treatment and kept in 20 by 20 by 20 cm Plexiglas cages. Three days after the bioassay, 50 adults of each treatment were transferred to individual plastic cages with those designated for females bearing an oviposition substrate. Dead males and females, as well as the number of eggs laid were recorded daily. Control adults (n = 30 for each treatment) were treated with plain acetone and kept/tested likewise. Thus, any differences in survival between treatment and control flies were attributed to the effects of limonene alone.

### Statistical analyses

Data analyses were performed using SPSS 22.0 (SPSS Inc., Chicago, IL, U.S.A.). The concentration-mortality data were analyzed using probit analysis. Probit-transformed mortality was regressed against Log_10_-transformed dose. LD_50_ and LD_90_ values, 95% confidence intervals and slopes of the regression lines were calculated for all treatments. No statistically significant mortality was observed in controls, therefore mortality percentages were not corrected. The *χ*^2^ value was used to measure the goodness-of-fit of the probit regression equation. The lifespan of males and females that survived exposure to limonene LD_20_ was assessed by the Kaplan Meier estimator and comparisons were conducted using the log rank (Mantel—Cox) test. The effect of diet and exposure to limonene LD_20_ on female fecundity was estimated using the Generalized Linear Models (GLMs) with linear error distribution. Pairwise comparisons of estimated marginal means were conducted using the Bonferroni method (*P* < 0.05).

## Results

### Acute toxicity

The toxicities of the three monoterpenes against male and female medflies that fed with yeast and sugar (YS) or with sugar only (S) were evaluated through topical application. Overall, females were more resistant than males to all three compounds regardless of the diet (Table 1). Females fed with YS were more sensitive to α-pinene followed by limonene and linalool as suggested by the non-overlapping 95% CI of LD_50_ values (Table 1). Moreover, females fed with S were more resistant to linalool. No significant differences were observed concerning the toxicity among the 3 compounds tested on males fed either with YS or with S only. The toxicity of each compound when assessed separately was not affected by the diet provided to males and females (S1 Dataset).

**Table 1:** LD_50_ and LD_90_ values for three monoterpenes (limonene, α-pinene and linalool) topically applied to male and female medflies fed with yeast and sugar (YS) or with sugar only (S).

nl/fly (95% CI^a^)						
Yeast Sugar		LD_50_	LD_90_	Slope ± SE	*χ*^2^^b^	df
males	Limonene	8.34 (2.48–13.14)	44.01 (31.35–91.67)	1.77 ± 0.46	5.54	4
	α-Pinene	7.71 (2.59–11.75)	30.34 (22.13–54.28)	2.15 ± 0.55	2.23	3
	Linalool	10.37 (3.79–15.52)	57.05(39.45–136.83)	1.73 ± 0.44	1.37	4
females	Limonene	31.72 (22.44–43.23)	155.77 (89.78–678.94)	1.85 ± 0.45	5.05	3
	α-Pinene	17.20 (10.91–22.33)	71.32 (50.51–146.92)	2.07 ± 0.43	2.82	4
	Linalool	49.39 (38.43–77.90)	210.42 (114.05–1056.22)	2.04 ± 0.48	1.72	4
**Sugar**						
males	Limonene	9.11 (3.61–13.54)	39.74 (29.40–69.86)	2.00 ± 0.47	6.11	4
	α-Pinene	11.21 (0.47–18.95)	40.17 (24.63–379.60)	2.31 ± 0.48	7.70	4
	Linalool	7.81 (2.26–12.29)	36.84 (26.06–76.85)	1.90 ± 0.51	1.35	3
females	Limonene	25.20 (19.97–30.43)	75.51 (56.63–128.20)	2.69 ± 0.47	4.68	4
	α-Pinene	25.30 (20.19–32.26)	69.78 (48.98–148.64)	2.91 ± 0.59	2.46	2
	Linalool	41.93 (31.39–67.28)	204.89 (105.91–1249.32)	1.86 ± 0.46	2.94	3

^a^ LD_50_ or LD_90_ values are considered significantly different when 95% CI fail to overlap.

^b^ *χ*^2^ goodness-of-fit test (all P≥0.05).

### Effect of limonene sub-lethal dose (LD_20_) and diet on adult demographic traits

Overall males outlived females regardless of the diet regime and the exposure to limonene LD_20_ (Wald test *t* = 39.897, d.f. = 1, *P* < 0.001) (Table 2). Adults fed with YS lived longer than those fed with S (Wald test *t* = 20.739, d.f. = 1, *P* < 0.001) regardless of being subjected to limonene LD_20_ or not. Exposure to limonene LD_20_ significantly enhanced adult survival regardless of the diet regime (Wald test *t* = 9.019, d.f. = 1, *P* < 0.05). None of the two way interactions among the 3 factors (sex, diet and treatment with Limonene LD_20_ or Acetone) was significant (Wald test *t*_diet*sex_ = 2.294, *t*_diet*treatment_ = 2.707, *t*_sex*treatment_ = 0.523, d.f. = 1, *P* > 0.05).

**Table 2:** Mean lifespan and percentiles of medflies subjected to acetone (control) and to sub-lethal dose LD_20_ of limonene.

Acetone (control)		Mean lifespan (days ± SE)	Quartiles (days)		
			25	50	75
males	S (n = 30)	21.63 ± 1.38	27 ± 1.39	20 ± 2.05	17 ± 2.51
	YS (n = 30)	31.10 ± 2.23	38 ± 2.90	28 ± 3.13	21 ± 3.23
females	S (n = 29)	13.41 ± 1.81	17 ± 3.23	11 ± 2.69	5 ± 1.20
	YS (n = 29)	20.38 ± 1.71	26 ± 1.31	18 ± 1.34	13 ± 1.81
**Limonene**					
males	S (n = 50)	26.92 ± 1.00	31 ± 1.81	28 ± 0.69	22 ± 1.72
	YS (n = 50)	33.74 ± 1.66	42 ± 2.44	34 ± 1.96	25 ± 2.22
females	S (n = 49)	19.49 ± 1.50	27 ± 2.43	18 ± 1.17	11 ± 0.81
	YS (n = 49)	21.94 ± 1.58	23 ± 3.01	19 ± 0.58	16 ± 0.73

**Fig 1:**
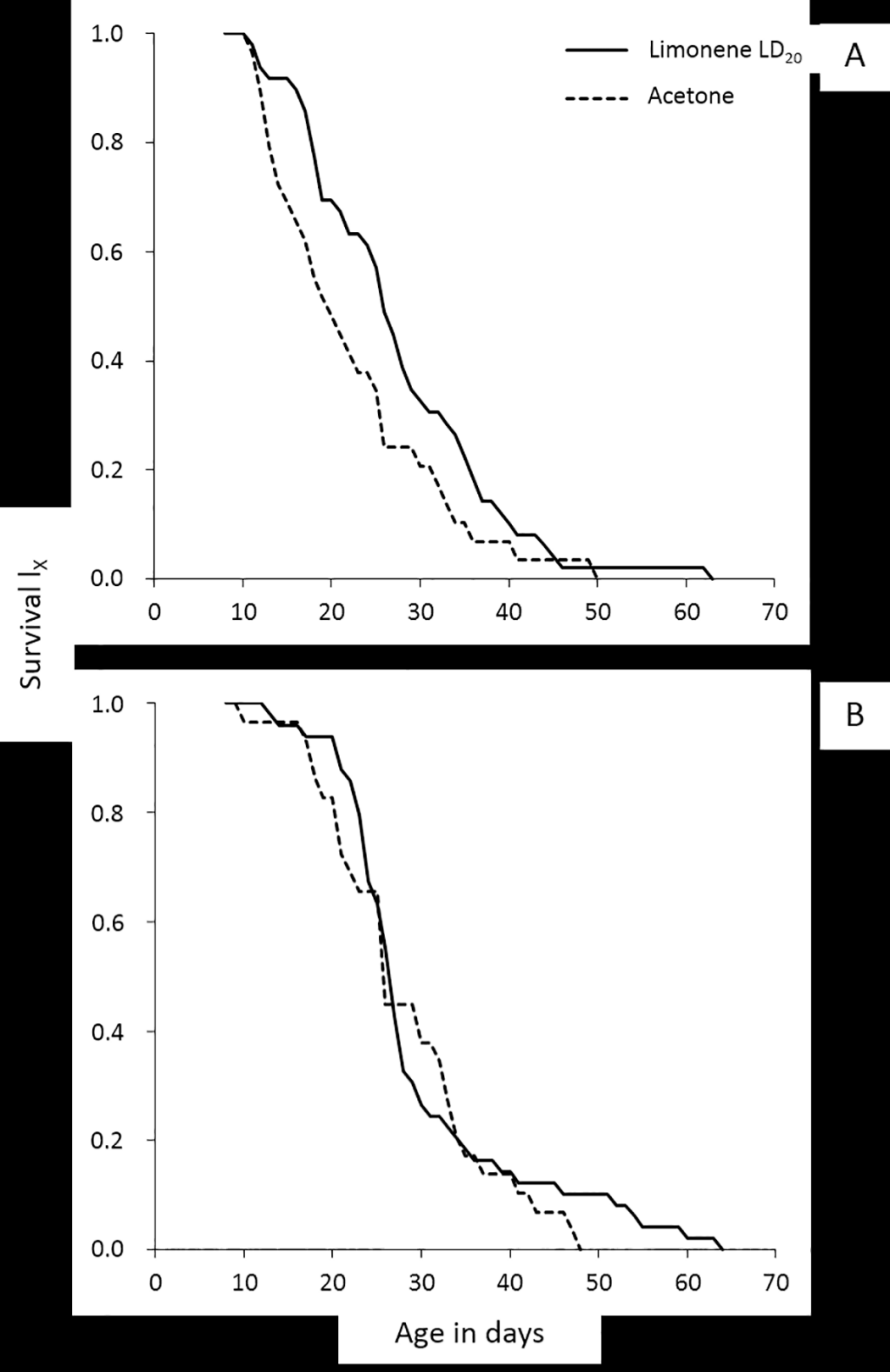
Age-specific survival curves for females that fed with S (A) or YS (B) and were subjected to limonene LD_20_ or to pure acetone (control).

**Fig 2:**
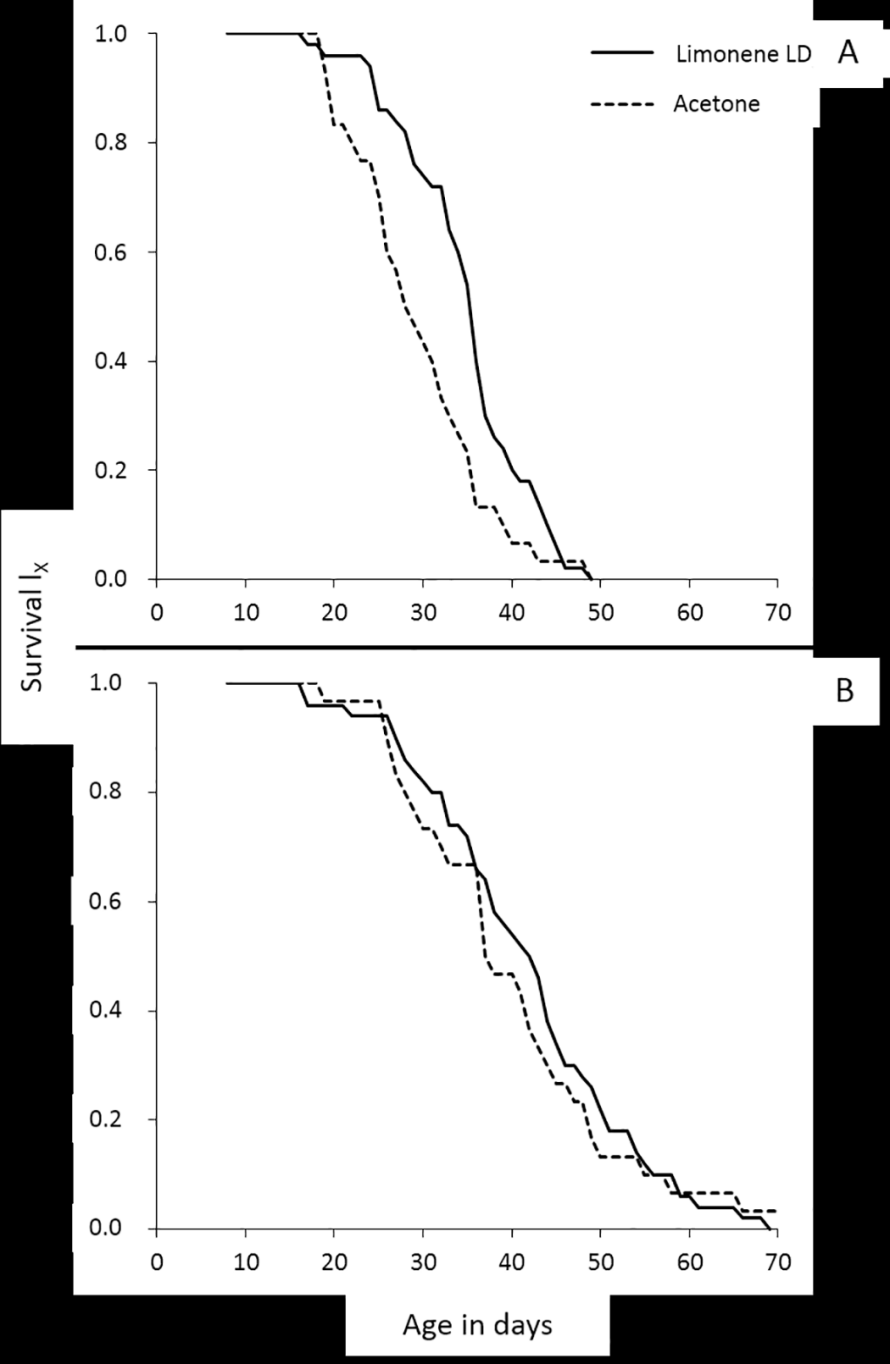
Age-specific survival curves for males that fed with S (A) or YS (B) and were subjected to limonene LD_20_ or to pure acetone (control).

Differences in survival between exposed and non-exposed to limonene LD_20_ adults, were tested for each sex and diet regime separately. Sugar fed females lived significantly longer when subjected to limonene than control ones (Wald test *t* = 4.595, d.f. = 1, *P* < 0.05) (Fig 1A), but the longevity of exposed and non-exposed to limonene females that fed with YS did not differ (Wald test *t* = 0.299, d.f. = 1, *P* > 0.05) (Fig 1B). Likewise, males fed with S lived significantly longer when subjected to limonene than control males (Wald test *t* = 6.059, d.f. = 1, *P* < 0.05) (Fig 2A). On the contrary, no significant differences in longevity were observed between exposed to limonene and control males that fed with YS (Wald test *t* = 0.513, d.f. = 1, *P* > 0.05) (Fig 2B).

**Fig 3:**
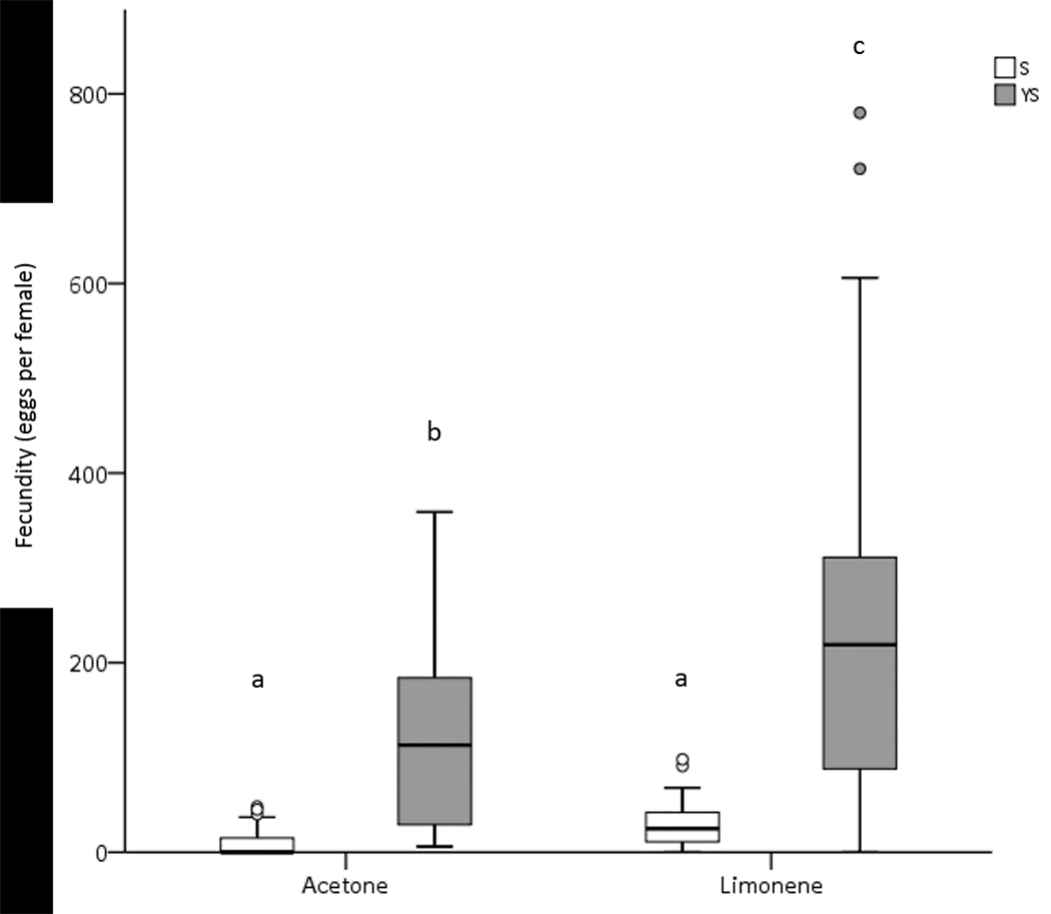
Box plots depicting fecundity distributions of females fed with either S or YS that were exposed to acetone (control) and to limonene LD_20_. Different letters indicate significant differences among female cohorts (Bonferroni adjustment, P < 0.001 in all cases)

Female lifetime fecundity was significantly affected by the diet (squared Wald test *χ*^*2*^ = 75.681, d.f. = 1, *P* < 0.001), the exposure to limonene LD_20_ (squared Wald test *χ*^*2*^ = 10.072, d.f. = 1, *P* < 0.05) and by the interaction of the above factors (squared Wald test *χ*^*2*^ = 4.892, d.f. = 1, *P* < 0.05) indicating a differential response of female fecundity to the exposure to limonene within the two diets. Yeast sugar fed females exposed to limonene LD_20_ laid significantly more eggs than control ones. Although there was a similar trend for the S fed females, no significant differences were detected. The fecundity of females fed with S was significantly lower than of females fed with YS regardless of the exposure to limonene (Fig 3, S1 Dataset).

## Discussion

Through our laboratory trials, we extended the range of effective EO compounds against medfly adults and we determined the toxicities of three different constituents of citrus EOs on male and female medflies that had access either to a YS or to a S only diet. Overall, females are more resistant than males to all three compounds regardless of the diet. Limonene LD_20_ enhances the survival of adult medflies when they are deprived of protein. Fecundity is positively affected when females are exposed to limonene sub-lethal dose. Both these latter findings indicate a hormetic-like effect of limonene to adult medflies.

The three monoterpenes tested had similar toxicities to adult medflies. An earlier study regarding the toxicity of these compounds on medfly larvae suggests that limonene and linalool exhibit higher toxicity than of that of α-pinene [14]. This discrepancy regarding variability in toxicity among the three compounds could be attributed to the different modes of application of EO compounds (contact ingestion vs topical application) as well as to the different developmental stages of flies tested (larvae vs adults). High toxicity of pinenes has been demonstrated in other studies. For example, Benelli and co-workers [21] showed that a dose of 0.25 μl/fly of *Thuja occidentalis* EOs with α-pinene as one of their dominant components (27.7%) causes a 96.7% mortality on adult medflies when applied topically. Towards the same direction, high insecticidal activity has been indicated for α- and β-pinene on Colorado potato beetle *Leptinotarsa decemlineata* Say (Coleoptera: Chrysomelidae) larvae and adults [25]. In the same study α-pinene was found to be more toxic to adult beetles than larvae.

Interestingly, our results show that α-pinene exhibits a significantly higher toxicity only to females fed with YS compared to the other two compounds. Toxic effects of α-pinene should further be explored in relation to diet and sex on medfly and other tephritid species. In a recent study Gerofotis and co-workers [26] showed that α-pinene induces sex-specific effects on demographic traits of adult olive flies (*Bactrocera oleae*) that are more pronounced in dietary restricted conditions. Specifically, despite the stimulatory effect of α-pinene’s aroma to the longevity of male *B*. *oleae* no effects were observed in the case of females. This could be a first indication of a differential influence of this compound to male and female Tephritids.

Our study clearly shows that males are more susceptible than females to the toxicity of the three compounds regardless of the dietary conditions. Previous studies report such sex differences regarding toxicity of Lamiaceae and Myrtaceae EOs on *Acanthoscelides obtectus* (Say) (Coleoptera: Chrysomelidae) [27] and of insecticides on the parasitoid *Diglyphus begini* (Ashmead) (Hymenoptera: Eulophidae) [28] that are attributed to variability in adult size. Hence, this is not a surprising outcome since female medflies are larger (YS: 9.27±0.14 mg, S: 8.18±0.11 mg) than males (YS: 6.65±0.13 mg, S: 6.60±0.10 mg) and as a result the same amount of the solution applied on the abdomen is dispersed and absorbed by a wider surface and body mass. Other factors that may account for this difference in mortality between the two sexes are metabolism and behavior. The uptake of the compound may vary between the two sexes due to differences in toxicokinetics such as absorbance, distribution to target and non-target sites, breakdown and excretion [29]. To our knowledge, no information is available regarding sex differences of EOs toxicity in medfly or any other species of Diptera. Further exploitation of sex specific physiological mechanisms involved in EOs detoxification pathways should shed important light towards this direction.

The positive effects of limonene sub-lethal doses on adults’ fitness compared to controls could be the outcome of either a “selection effect” or a hormetic-like effect. The fact that treated adults (both males and females) lived significantly longer only in the case of the sugar-only diet points towards rejecting the “selection effect” hypothesis supporting a possible hormetic-like effect. The concept of hormesis defines a “dose–response phenomenon characterized by a reversal of the response between low and high doses of a stressor” [30]. Concerning insects, the stress-inducing factor is usually an insecticide and less often radiation, temperature or oxidative stress. Lately, growing evidence supports that phytochemicals may act as hormetic factors to heterotrophic organisms, including insects, by modulating evolutionary conserved signaling pathways [6]. Here we address the stimulatory effect to medfly lifespan of low (sub-lethal) doses of limonene that is toxic at higher doses. Though our results indicate that all three compounds induce high insecticidal toxicity, sub-lethal exposure to limonene (the predominant constituent of citrus EOs) results in stimulatory responses of the lifetime survival of adult medflies with access to a poor nutritional environment (S only diet). Thus, our study establishes for the first time a limonene-induced hormetic-like response. Both males and females exposed to limonene sub-lethal dose exhibit extended longevity compared to control flies and this response is more pronounced in a nutritionally poor environment. Previous studies regarding the effect of plant extracts on longevity of Tephritids are in agreement with our findings [26, 31, 32]. Mexican fruit fly adults (*Anastrepha ludens*) live longer when provided an oregano-cranberry mixture and this result is more obvious in protein restricted conditions [31]. Likewise, exposure to the aroma of α-pinene increases longevity of male *B*. *oleae* only in dietary restricted conditions [26]. The interaction of carbohydrate and protein equilibrium with plant extracts, such as EOs and their compounds, and the way lifespan is modulated has not been elucidated so far. Although the mode of application of phytochemicals (dietary supplement, topical application, gas or aroma exposure) plays a crucial role as different pathways (nutrient-, energy-, stress-sensing) within the organism can be triggered, lifespan extension is often observed as a common provoked effect [6]. Contact interaction with limonene sub-lethal dose could affect the homeostatic mechanisms that regulate adult lifespan through hormone and neurotransmitter alteration (discussed in [26]). Nevertheless, future studies should exploit and elaborate the underlying physiological and/or molecular mechanisms activated during contact exposure to this plant derived compound.

The present study provides clear evidence of a stimulating effect of limonene sub-lethal dose to medfly fecundity especially in a highly nutritional environment. This finding is consistent with Ioannou et al. [2] who have tested the stimulatory effects of the aroma of limonene on medfly fecundity. Interestingly, the topical application of limonene LD_20_ on female abdomen, including the ovipositor, produces a similar response. The ovipositor (and/or abdomen) in Tephritids is equipped with chemoreceptors that facilitate females in finding the adequate oviposition site to lay their eggs [33] in order to increase the probability of offspring to survive. In addition, limonene is the most abundant component of the majority of citrus essential oils and may be detected by females when inserting their ovipositor into the peel of citrus fruits. The positive effect of limonene sub-lethal dose expressed as increased fecundity rates may indicate a hormetic-like effect of this component, as well. To the extent of that, similar responses have been recorded when low (0–1 μl) versus high (> 1 μl) doses of sweet orange EO were offered into artificial oviposition substrates to female medflies [2]. Low amount of sweet orange EO had a stimulatory effect by increasing fecundity and decreasing preoviposition periods while the opposite was observed with high doses of sweet orange EO. Hormetic-like effects of phytochemicals and insecticides have been recorded as increased fecundity in other insect species, as well [5, 30, 34]. Azadirachtin sub-lethal doses increase the fecundity of *Zabrotes subfasciatus* (Boheman) (Chrysomelidae: Bruchinae) and decrease the survival [34], possibly indicating a trade-off between longevity and reproduction in stressful environments that is induced by either an increase of juvenile hormone levels [35] or a shift in resource allocation [36]. In the present study, female medflies that feed with YS increase their fecundity with no significant alterations in survival when exposed to limonene sub-lethal doses. Although the mechanisms regulating hormesis induced by phytochemicals are not well understood, it is plausible to argue that the increased fecundity accompanied by increased longevity patterns may be attributed to hormonal alterations or energetic trade-offs, as well. However, exploitation of the underling physiological and molecular mechanisms participating in these circumstances is crucial to clarify survival versus reproduction equilibrium under a hormesis-induced environment.

The present study provides additional data regarding the interaction of medfly with citrus host species and introduces a hormetic-like response of medfly to the most abundant compound of citrus EOs. The toxicity induced by several citrus EOs compounds through contact and absorbance by adult medflies could provide useful insights towards the development of novel control tools (encapsulated EO compounds in food baits or insecticides). Nevertheless, additional knowledge concerning the induction of hormetic-like responses by specific citrus EOs compounds and the interaction with diet and sex should be explored.

## Supporting information

S1 Dataset Excel file with the raw data used in this study. (XLSX)
